# Different RNA recognition by ProQ and FinO depends on the sequence surrounding intrinsic terminator hairpins

**DOI:** 10.1261/rna.080206.124

**Published:** 2025-05

**Authors:** Maria D. Mamońska, Maciej M. Basczok, Ewa M. Stein, Julia Kurzawska, Mikołaj Olejniczak

**Affiliations:** Institute of Molecular Biology and Biotechnology, Faculty of Biology, Adam Mickiewicz University, Uniwersytetu Poznańskiego 6, 61-614 Poznań, Poland

**Keywords:** ProQ, FinO, FinO domain, sRNA, bacterial regulatory RNA

## Abstract

*Escherichia coli* ProQ and FinO proteins both have RNA-binding FinO domains, which bind to intrinsic transcription terminators, but each protein recognizes distinct RNAs. To explore how ProQ and FinO discriminate between RNAs, we transplanted sequences surrounding terminator hairpins between RNAs specific for each protein, and compared their binding to ProQ, the isolated FinO domain of ProQ (ProQ^NTD^), and FinO. The results showed that the binding specificity of chimeric RNAs toward ProQ, ProQ^NTD^, or FinO was determined by the origin of the transplanted sequence. Further analysis showed that the sequence surrounding the terminator hairpin, including a purine–purine mismatch, in natural RNA ligands of FinO and in chimeric RNAs, weakened their binding by ProQ^NTD^. Overall, our studies suggest that RNA sequence elements surrounding the intrinsic terminator hairpin contribute to the discrimination between RNAs by ProQ and FinO.

## INTRODUCTION

RNA-binding proteins play important roles in gene expression regulation dependent on small RNAs (sRNAs) in bacteria (Wagner and Romby 2015; Gorski et al. 2017; Holmqvist and Vogel 2018; Hor et al. 2020). A well-studied example is the matchmaker protein Hfq, which promotes interactions between sRNAs and messenger RNAs (mRNAs), rearranges RNA structure, and affects RNA stability (Soper and Woodson 2008; Schu et al. 2015; Updegrove et al. 2016; Andrade et al. 2018; Kavita et al. 2018; Kwiatkowska et al. 2018; Roca et al. 2022; Malecka and Woodson 2024). Another example is FinO-domain proteins, which are present alongside Hfq in numerous β- and γ-proteobacteria, including species important for human health (Glover et al. 2015; Attaiech et al. 2017; Olejniczak and Storz 2017; Holmqvist et al. 2020). The FinO-domain proteins are involved in diverse physiological processes, such as plasmid conjugation (Glover et al. 2015), natural transformation (Attaiech et al. 2016), osmoregulation (Kerr et al. 2014; Melamed et al. 2020), adaptation to available nutrients (El Mouali et al. 2021b; Katz et al. 2021), flagellar assembly (Rizvanovic et al. 2021), persister cells formation (Rizvanovic et al. 2022), and virulence (Westermann et al. 2019; Bergman et al. 2024). However, the molecular mechanisms of the contributions of the FinO-domain proteins to these processes are not fully understood.

The FinO-domain proteins consist of the core FinO domain and additional N- or C-terminal extensions (Glover et al. 2015; Attaiech et al. 2017; Olejniczak and Storz 2017; Holmqvist et al. 2020). Despite limited sequence identity, the FinO domains from different proteins have similar overall structure consisting of five α-helical segments (Ghetu et al. 2000; Chaulk et al. 2010; Gonzalez et al. 2017; Immer et al. 2020; Kim et al. 2022). Several studies showed that a FinO domain is the part of these proteins that specifically recognizes those RNA molecules, which contain intrinsic transcription terminators (Ghetu et al. 2002; Chaulk et al. 2010, 2011; Attaiech et al. 2016; Gonzalez et al. 2017; Bauriedl et al. 2020; Pandey et al. 2020; Stein et al. 2020; Kim et al. 2022). On the other hand, the C-terminal extensions of *Legionella pneumophila* RocC and *Salmonella enterica* ProQ are important for their physiological functions (Attaiech et al. 2016; El Mouali et al. 2021b; Rizvanovic et al. 2021), and the C-terminal extension of *Escherichia coli* ProQ may also contribute to nonspecific RNA binding (Gonzalez et al. 2017; Stein et al. 2020).

The intrinsic transcription terminators constitute the binding sites of FinO-domain proteins in their RNA ligands. The Grad-seq study, which identified several hundred RNAs bound by ProQ in *S. enterica*, showed that ProQ recognizes structured motifs in bound RNAs (Smirnov et al. 2016). Further global profiling studies of RNA binding by *S. enterica* and *E. coli* ProQ using CLIP-seq (Holmqvist et al. 2018), by *E. coli* ProQ using RIL-seq (Melamed et al. 2020), and by *Neisseria meningitidis* minimal ProQ (NMB1681) using CLIP-seq (Bauriedl et al. 2020) showed that typical RNA-binding sites of ProQ were GC-rich and followed by uridine-rich sequences, which is consistent with intrinsic transcription terminators. The studies using purified components also showed that F-like plasmid FinO protein, *L. pneumophila* RocC, *N. meningitidis* ProQ, and *E. coli* ProQ bound tightly to intrinsic terminator hairpins with adjacent single-stranded regions, which are derived from their native RNA ligands (Jerome and Frost 1999; Attaiech et al. 2016; Stein et al. 2023; Basczok and Olejniczak 2025). Additionally, it was observed that the RNAs bound by ProQ contained an A-rich sequence motif on the 5′ side of the terminator that prevented their binding by Hfq, which is another global RNA-binding protein in *E. coli* (Stein et al. 2020). Further studies showed a more detailed picture of the recognition of the terminator structures by FinO-domain proteins. The recent X-ray study showed that the FinO domain of *L. pneumophila* RocC protein recognized the lower part of the stem of the terminator hairpin, and its single-stranded 3′-terminal tail (Kim et al. 2022). It was observed that the F-like plasmid FinO protein also recognized the lower part of the terminator of FinP RNA (Arthur et al. 2011) and the 3′ tail (Jerome and Frost 1999). Additionally, the contribution of the stem of the terminator hairpin to RNA binding by *S. enterica* and *E. coli* ProQ was shown using mutations disrupting or shortening this region (Holmqvist et al. 2018; Stein et al. 2020), and the contribution of the sequences surrounding the terminator was shown using truncation experiments (Stein et al. 2020, 2023).

The RNA-binding site is located on the concave face of the FinO domain (Ghetu et al. 2002; Pandey et al. 2020; Kim et al. 2022; Stein et al. 2023). The recent X-ray crystallography study showed that the terminator hairpin of RocR RNA is bound on the concave face of the FinO domain of *L. pneumophila* RocC protein (Kim et al. 2022). In this site several residues of the N-terminal part of α-helix 5 contact the double-stranded base of the terminator hairpin, while other conserved residues form hydrogen bonds with terminal nucleotides of the 3′ polypyrimidine tail (Kim et al. 2022). The RNA interactions with the FinO domain were first shown directly using the cross-linking of the F-like plasmid FinO protein binding to a fragment of FinP RNA, which revealed contacts including arginine and lysine residues on the concave face of the FinO domain (Ghetu et al. 2002). The involvement of the FinO domain of *E. coli* ProQ in RNA binding was also supported by hydrogen-deuterium exchange studies (Gonzalez et al. 2017). Additionally, the concave face of the FinO domain of *E. coli* ProQ was indicated as the RNA-binding site by analyzing the binding of the ProQ mutants in bacterial cells using bacterial three-hybrid assay (Pandey et al. 2020), and in vitro using gelshift assay (Stein et al. 2023). Substitutions of several amino acids on the concave face of the FinO domains of *S. enteric*a ProQ and *L. pneumophila* RocC were also identified by mutagenesis studies exploring the physiological outcomes of the mutations, which confirms their essential importance for the function of FinO-domain proteins (Attaiech et al. 2016; El Mouali et al. 2021b; Rizvanovic et al. 2021).

The FinO-domain proteins recognize specific sets of RNAs in bacterial cells (Attaiech et al. 2016; Smirnov et al. 2016; Holmqvist et al. 2018; Bauriedl et al. 2020; Gerovac et al. 2020; Melamed et al. 2020; El Mouali et al. 2021a). Some FinO-domain proteins bind hundreds of RNAs (Smirnov et al. 2016; Bauriedl et al. 2020; Holmqvist et al. 2020; Melamed et al. 2020), while others bind just a few RNAs (Attaiech et al. 2016; Gerovac et al. 2020; El Mouali et al. 2021a). Interestingly, *E. coli* and *S. enterica* have both a global RNA-binding protein ProQ, which binds numerous RNAs, including regulatory RNAs and mRNAs (Smirnov et al. 2016; Holmqvist et al. 2018; Melamed et al. 2020), and a narrow-specificity RNA-binding protein FinO, which binds only two regulatory RNAs, FinP and RepX (El Mouali et al. 2021a). The chromosomally encoded ProQ protein is composed of the N-terminal FinO domain, a positively charged linker, and the C-terminal Tudor domain (Smith et al. 2004, 2007; Chaulk et al. 2011; Gonzalez et al. 2017). The F-like plasmid-encoded FinO protein consists of the FinO domain accompanied by a positively charged N-terminal extension (Ghetu et al. 2000). Because both ProQ and FinO recognize intrinsic transcription terminators as their main binding motifs in RNAs (Jerome and Frost 1999; Arthur et al. 2011; Holmqvist et al. 2018; Melamed et al. 2020; Stein et al. 2020), it is not clear how their specificity of RNA recognition is determined.

To find out how ProQ and FinO recognize their respective RNA ligands, we transplanted sequence motifs surrounding intrinsic terminator hairpins between RNAs specific for each protein and measured how it affected their binding affinities to each protein. The results of these experiments provided new insights into the role of RNA sequences adjacent to intrinsic terminators in enabling the discrimination between RNAs by ProQ and FinO.

## RESULTS

### Comparison of the 3′-terminal sequences of top RNA ligands of ProQ and FinO

To better understand how ProQ and FinO distinguish between preferred RNAs, we analyzed the sequences and structures of top RNA ligands of each protein (Supplemental Figs. S1–S3), which were previously identified using global profiling methods (Holmqvist et al. 2018; Melamed et al. 2020; El Mouali et al. 2021a). We used RNAstructure software to compare the sequences and secondary structures of this region for two sets of top 20 RNAs bound by ProQ, which were identified by CLIP-seq or RIL-seq in *E. coli* (Holmqvist et al. 2018; Melamed et al. 2020), as well as for *E. coli* FinP and *S. enterica* RepX, which are the ligands of F-like plasmid FinO protein (Supplemental Figs. S1–S3; van Biesen and Frost 1994; Jerome and Frost 1999; El Mouali et al. 2021a). Because previous studies showed that FinO domains bind RNAs at the site consisting of the lower part of the intrinsic terminator hairpin and surrounding sequence (Arthur et al. 2011; Stein et al. 2020; Kim et al. 2022), we predicted the structures of RNA fragments consisting of the terminator hairpin, the 10 nt upstream region, and the 3′ tail (Supplemental Figs. S1–S3). In this analysis, the lower end of the terminator hairpin was defined as the closing G-C or C-G base pair of the hairpin, which was directly adjacent to the 3′ tail consisting mainly of U residues.

At first, we compared the sequences of the three base pairs at the base of the terminator hairpins in the RNA ligands of ProQ and FinO. Among the 33 unique RNAs identified by RIL-seq or CLIP-seq as the top 20 ligands of ProQ (Holmqvist et al. 2018; Melamed et al. 2020), most have only G-C or C-G base pairs in all three lowest base pairs of the terminator hairpin (Supplemental Figs. S1, S2). Similarly, in the two RNAs bound by FinO, RepX, and FinP, which were identified by RIP-seq (El Mouali et al. 2021a), the three lowest base pairs consisted either mostly or only of G-C or C-G pairs (Supplemental Fig. S3). The fact that similar sequences are present in this part of the hairpin in RNA ligands of ProQ and FinO suggests that this region is not essential to discriminate between RNAs by these proteins.

Next, we compared the secondary structures formed by the 3′ tails and sequences upstream of the terminator hairpins. We found that in 28 of the 33 unique RNA ligands of ProQ at least 2 nt of the 3′ tail closest to the base of the hairpin were involved in base-pairing with the opposing nucleotides upstream of the hairpin (Supplemental Figs. S1, S2). In contrast, neither of the RNA ligands specific to FinO showed base-pairing between the nucleotides of the 3′ tail and the opposing nucleotides upstream of the hairpin (Supplemental Fig. S3). This difference suggests that the region just below the closing base pair of the terminator hairpin could be involved in the distinct recognition of RNAs by ProQ and FinO.

Finally, we analyzed the nucleotide composition at the two positions of the 3′ tail and the two positions of the upstream sequence, which were closest to the base of the terminator hairpin. Among those ProQ-specific RNAs, in which these nucleotides were base-paired, most often A-U base pairs, and less often U-A or G-U base pairs were present, while in those RNAs, in which these nucleotides were unpaired, only pyrimidine–pyrimidine mismatches—C-U, U-U, or C-C—were present in these two positions (Supplemental Figs. S1, S2). In contrast, in FinO-specific FinP and RepX RNAs, the closing base pair of the hairpin was neighbored by a purine–purine mismatch, the A-G mismatch, followed by either C-A or U-C mismatch (Supplemental Fig. S3). In summary, in the two positions directly below the closing base pair of the terminator hairpin the top RNAs bound by ProQ have either canonical A-U or G-U base pairs or pyrimidine–pyrimidine mismatches, while purine–purine mismatches are not found in these positions. On the other hand, both RNA ligands of FinO contain a purine–purine mismatch at the first position below the terminator hairpin. The fact that the sequences immediately adjacent to the closing base pair of the terminator hairpin are different between RNA ligands of ProQ and FinO, in a way which could affect local RNA structure, suggests that these sequence elements could contribute to differential RNA recognition by these two proteins.

### *malM*-3′ RNA is specifically recognized by the ProQ protein, and FinP RNA is specifically recognized by the FinO protein

**FIGURE 1.**
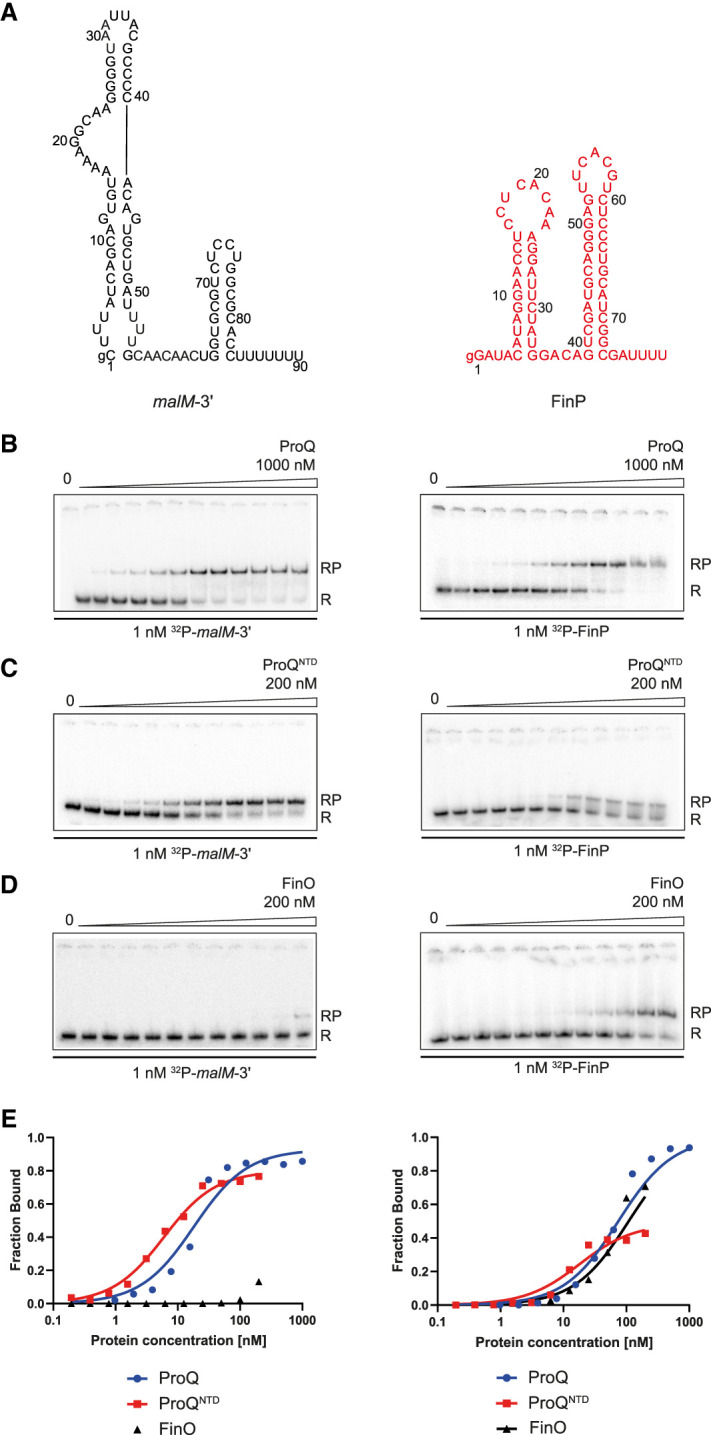
Comparison of *malM*-3′ and FinP RNA binding to ProQ, ProQ^NTD^, and FinO. (*A*) Secondary structures of *malM*-3′ (black font) and FinP RNAs (red font), which were predicted using RNAstructure software (Reuter and Mathews 2010). The lowercase g denotes guanosine residue added on 5′ ends of RNA molecules to enable T7 RNA polymerase transcription. (*B*–*D*) The gelshift analysis of *malM*-3′ and FinP binding to full-length ProQ (*B*), ProQ^NTD^ (*C*), and FinO (*D*). Free ^32^P-labeled RNA is marked as R and RNA-protein complexes as RP. (*E*) The plots of fraction-bound data versus protein concentration from *B* to *D* are shown. The fitting of the quadratic equation into *malM*-3′ binding data provided a *K*_d_ value of 18 nM for binding to ProQ, and 5.5 nM for binding to ProQ^NTD^, while the *K*_d_ value for binding to FinO was estimated as higher than 200 nM. The fitting of the quadratic equation into FinP binding data provided a *K*_d_ value of 74 nM for binding to ProQ, 18 nM for binding to ProQ^NTD^, and 109 nM for binding to FinO. The average equilibrium dissociation constant (*K*_d_) values calculated from at least three independent experiments are shown in Table 1.

To test if ProQ and FinO distinctly bind their natural RNA ligands, we used a gelshift assay to compare the binding of each purified protein to two RNAs that were previously identified using global profiling as their natural ligands in *E. coli* cells (Fig. 1A; Holmqvist et al. 2018; Melamed et al. 2020). One of these RNAs was the 3′ UTR of *malM* mRNA (*malM*-3′), which is the top RNA bound by *E. coli* ProQ identified using the RIL-seq method (Melamed et al. 2020), and one of the top ligands of ProQ identified using CLIP-seq method (Supplemental Figs. S1, S2; Holmqvist et al. 2018). The in vitro binding of *malM*-3′ to *E. coli* ProQ and ProQ^NTD^ has already been studied (Stein et al. 2020). The other of these RNAs was FinP RNA, which is the main RNA bound by FinO protein in *E. coli* and *S. enterica* (Supplemental Fig. S3; van Biesen and Frost 1994; El Mouali et al. 2021a). It was previously shown that full-length ProQ bound similarly tightly to RNAs containing intrinsic terminators and to RNAs without such structures, which suggested that there is a nonspecific component to RNA binding by the full-length ProQ (Stein et al. 2020). On the other hand, the isolated FinO domain of ProQ specifically recognized RNAs ending with intrinsic transcription terminators (Chaulk et al. 2011; Pandey et al. 2020; Stein et al. 2020). For that reason, we compared the binding of *malM*-3′ and FinP to the full-length ProQ and to the isolated FinO domain of ProQ (ProQ^NTD^).

At first, we compared the binding of full-length ProQ to both RNAs (Fig. 1B,E; Table 1; Supplemental Figs. S4, S5). The data showed that *malM*-3′ RNA, which is a natural ligand of ProQ, bound ProQ with a *K*_d_ value of 27 nM, while FinP, which is a natural ligand of FinO, bound ProQ threefold weaker. Next, we compared the binding of these RNAs to ProQ^NTD^ (Fig. 1C,E; Table 1; Supplemental Figs. S4, S5). *malM*-3′ bound ProQ^NTD^ with a *K*_d_ value of 5.8 nM, while FinP bound ProQ^NTD^ with a *K*_d_ value, which was about threefold weaker. Additionally, the fraction of FinP bound to ProQ^NTD^ at saturation was below 50%, while that of *malM*-3′ saturated at 80%, which further supports weaker binding of FinP to ProQ^NTD^. There could be several reasons for the low fraction of FinP bound to ProQ^NTD^ (Fig. 1C,E). One possibility is that complexes of FinP with ProQ^NTD^ are less stable and dissociate during electrophoresis. Another possibility is that the protein aggregates when it is in the presence of weaker binding RNAs. Alternatively, it could also suggest that an RNA forms an alternative conformation, which is not bound by the protein. Regardless of the detailed explanation for the lower fraction of FinP bound, these data show that FinP binds less well than *malM*-3′ to ProQ^NTD^. We also observed that the full-length ProQ bound its natural RNA ligand *malM*-3′ weaker than ProQ^NTD^ did. A similar difference was also observed in earlier studies of RNA binding by ProQ and ProQ^NTD^ (Chaulk et al. 2011), and could suggest that the long linker in full-length ProQ might be susceptible to misfolding in vitro leading to somewhat weaker RNA binding.

**TABLE 1.** Comparison of RNA binding to ProQ, ProQ^NTD^, and FinO proteins

^32^P-RNA	*K*_d_ (nM) (max. fraction bound %)		
	ProQ	ProQ^NTD^	FinO
*malM*-3′	27 ± 12 (91%)	5.8 ± 0.7 (80%)	>200 (15%)
FinP	79 ± 18 (85%)	19 ± 6 (44%)	110 ± 16 (76%)
*malM*-FinP	78 ± 22 (91%)	>200 (23%)	86 ± 17 (82%)
FinP-*malM*	25 ± 6.7 (79%)	5.3 ± 1.5 (62%)	280 ± 110 (57%)
*malM*-A-GU_6_	n.m.	1.9 ± 0.59 (56%)	150 ± 40 (59%)
*malM*-A-GAU_5_	160 ± 30 (64%)^a^	>200 (21%)	130 ± 18 (73%)
FinP-U-UAU_4_	n.m.	6.2 ± 3.1 (78%)	200 ± 73 (60%)
FinP-U-U_6_	37 ± 5.4 (75%)^a^	9.4 ± 4.5 (82%)	170 ± 45 (68%)
*cspE*81-3′	37 ± 7.1 (91%)^a^	2.4 ± 0.27 (85%)	>200 (10%)
*cspE*81-FinP	n.m.	4.9 ± 0.32 (61%)	88 ± 39 (74%)
*cspE*81-FinP-stem	130 ± 34 (85%)^a^	>200 (37%)	56 ± 16 (72%)
RepX	>1000 (29%)^a^	>200 (6%)	61 ± 23 (86%)
RepX-*malM*	n.m.	3.2 ± 1.6 (62%)	150 ± 67 (77%)
*malM*-RepX	61 ± 29 (67%)^a^	>200 (8%)	180 ± 93 (55%)

The *K*_d_ values were obtained by fitting the quadratic equation into binding data. The average *K*_d_ values with standard deviations were calculated from at least three independent experiments. When the fraction bound at the highest protein concentration used was lower than 40%, the *K*_d_ value was estimated as higher than 200 nM for FinO and ProQ^NTD^, or higher than 1000 nM for ProQ. (n.m.) Not measured.

^a^The *K*_d_ values are calculated based on the data provided in Supplemental Figure S9.

In the next step, we compared the binding of both RNAs to FinO (Fig. 1D,E; Table 1; Supplemental Figs. S4, S5). The fraction of *malM*-3′ bound to FinO protein was <20% at the maximum 200 nM concentration of FinO used, which allows estimating the *K*_d_ value as weaker than 200 nM. On the other hand, FinP bound FinO with the *K*_d_ value of 110 nM, while the binding saturated at more than 70% fraction bound, which confirms stronger binding of the FinO protein to its natural ligand FinP than to *malM*-3′ (Fig. 1D,E; Table 1). In summary, full-length ProQ, ProQ^NTD^, and FinO each have tighter binding affinity to its respective natural RNA ligand than to the other RNA, which is consistent with their distinct recognition of these RNAs in bacterial cells (Holmqvist et al. 2018; Melamed et al. 2020; El Mouali et al. 2021a).

### Transplanting sequence elements surrounding the terminator hairpin from FinP into *malM*-3′ switches the preferred binding from ProQ to FinO

**FIGURE 2.**
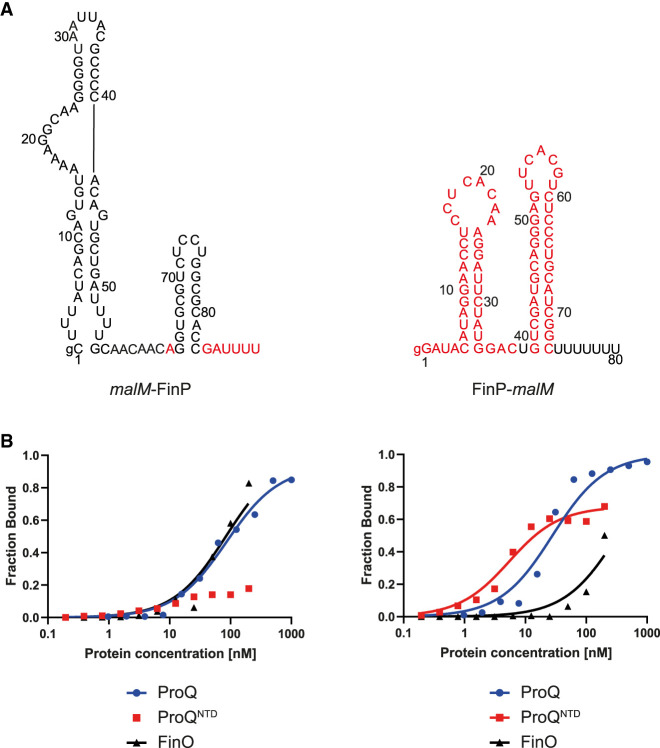
Comparison of *malM*-FinP and FinP-*malM* chimeras binding to ProQ, ProQ^NTD^, and FinO. (*A*) Secondary structures of *malM*-FinP and FinP-*malM* chimeras, which were predicted using RNAstructure software (Reuter and Mathews 2010). The sequences originating from *malM*-3′ are shown in black font and the sequences from FinP in red font. The lowercase g denotes guanosine residue added on 5′ ends of RNA molecules to enable T7 RNA polymerase transcription. (*B*) The respective binding data for ProQ, the ProQ^NTD^, and FinO are shown on the graphs *below* each RNA. The fitting of the quadratic equation into *malM*-FinP data provided a *K*_d_ value of 85 nM for binding to ProQ and 83 nM for binding to FinO, while the *K*_d_ value for binding to ProQ^NTD^ was estimated as higher than 200 nM. The fitting of the quadratic equation into FinP-*malM* data provided a *K*_d_ value of 27 nM for binding to ProQ, 4.9 nM for binding to ProQ^NTD^, and 327 nM for binding to FinO. Gels corresponding to the data in the plots are shown in Supplemental Figure S6. Average *K*_d_ values are shown in Table 1.

Because the analysis of sequences and secondary structures of RNA ligands of ProQ and FinO showed differences in the regions surrounding their terminator hairpins (Supplemental Figs. S1–S3), we designed chimeric constructs with these sequences transplanted between FinP and *malM*-3′ (Fig. 2) to test if transplanted sequences would affect their binding to ProQ, ProQ^NTD^, and FinO. In the natural sequence of *malM*-3′ the 3 nt 5′-adjacent to the terminator hairpin are ACU, and it has a 3′-terminal U_7_ tail (Fig. 1). The sequence elements transplanted from FinP into the *malM*-3′ body were the A residue 5′-adjacent to the terminator hairpin, and the 3′-terminal GAU_4_ sequence (Fig. 2A). The resulting *malM*-FinP chimera had the 3 nt 5′-adjacent to the terminator hairpin and the whole 3′-terminal tail the same as in FinP.

The data showed that *malM*-FinP chimera bound to full-length ProQ with similar affinity as FinP, and threefold weaker than *malM*-3′ (Fig. 2B; Table 1; Supplemental Fig. S6A). The binding of *malM*-FinP chimera to ProQ^NTD^ was even more strongly affected, because the fraction of *malM*-FinP bound was <25% at the maximum 200 nM concentration of ProQ^NTD^ used (Fig. 2B; Table 1; Supplemental Fig. S6B). Consistently, *malM*-FinP chimera bound to the FinO protein with similar affinity as FinP, and much more strongly than *malM*-3′ (Fig. 2B; Table 1; Supplemental Fig. S6C). In summary, these data indicated that the sequence elements of FinP transplanted into the body of *malM*-3′ strengthened the binding of resulting chimera to the FinO protein as compared to *malM*-3′, while they weakened its binding to either ProQ or ProQ^NTD^.

### Transplanting sequence elements surrounding terminator hairpin from *malM*-3′ into FinP switches the preferred binding from FinO to ProQ

To test if the corresponding sequence from *malM*-3′ can affect RNA recognition by ProQ and FinO, we constructed a chimera, in which the sequence elements from *malM*-3′ were transplanted into FinP RNA to create a FinP-*malM* chimera (Fig. 2A). In the natural sequence of FinP the 3 nt 5′-adjacent to the terminator hairpin are ACA, and there is a 3′-terminal GAU_4_ tail (Fig. 1A). The sequence elements from *malM*-3′ transplanted into the FinP body were the U residue 5′-adjacent to the terminator hairpin, and the 3′-terminal U_7_ tail (Fig. 2A). As a result, the FinP-*malM* chimera had the 3 nt 5′-adjacent to the terminator hairpin, and the whole 3′-terminal tail the same as in *malM*-3′.

The data showed that FinP-*malM* chimera bound ProQ similarly to *malM*-3′, and threefold tighter than FinP (Fig. 2B; Table 1; Supplemental Fig. S6A). Consistently, the FinP-*malM* chimera bound ProQ^NTD^ with the same affinity as *malM*-3′, and threefold stronger than FinP (Fig. 2B; Table 1; Supplemental Fig. S6B). On the other hand, FinP-*malM* bound the FinO protein twofold more weakly than FinP, but markedly stronger than *malM*-3′ (Fig. 2B; Table 1; Supplemental Fig. S6C). The fact that FinP-*malM* bound FinO stronger than *malM*-3′ could suggest that the structure context into which the sequence elements of *malM*-3′ were transplanted also affects the binding of FinO. Overall, these data showed that the sequence elements adjacent to the terminator hairpin in *malM*-3′ direct RNA recognition by the ProQ protein, even when they are placed in the context of an RNA that is not naturally bound by ProQ.

### Dissection of *malM*-3′ sequence elements, which determine the binding specificity toward ProQ

**FIGURE 3.**
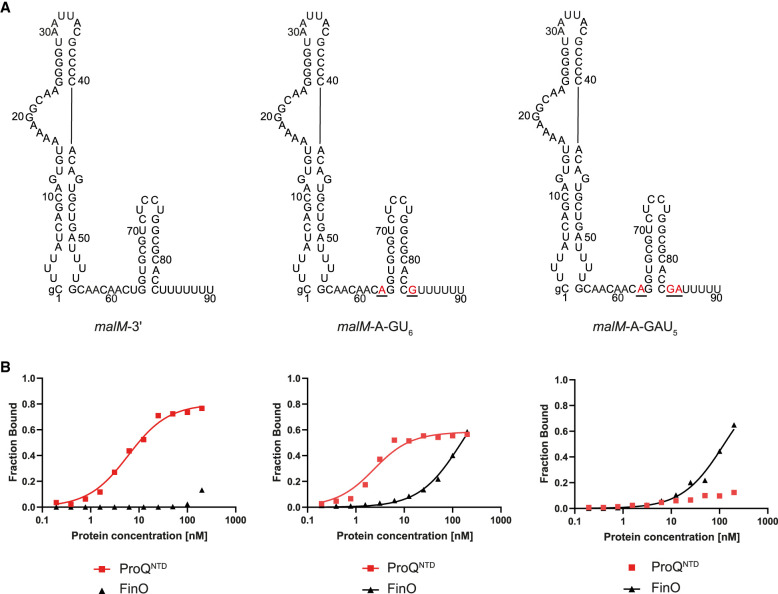
Comparison of *malM*-3′, *malM*-3′-A-GU_6_, and *malM*-3′-A-GAU_5_ binding to ProQ^NTD^, and to FinO. (*A*) Secondary structures of *malM*-3′, *malM*-3′-A-GU_6_, and *malM*-3′-A-GAU_5_, which were predicted using RNAstructure software (Reuter and Mathews 2010). The nucleotides from FinP which were substituted into *malM*-3′ are shown in red underlined font. The lowercase g denotes guanosine residue added on 5′ ends of RNA molecules to enable T7 RNA polymerase transcription. (*B*) The respective binding data for ProQ^NTD^ and FinO are shown on the graphs *below* each RNA. The fitting of the quadratic equation into *malM*-3′-A-GU_6_ data provided a *K*_d_ value of 1.8 nM for binding to ProQ^NTD^, and 151 nM for binding to FinO. The fitting of the quadratic equation into *malM*-3′-A-GAU_5_ data provided a *K*_d_ value of 122 nM for binding to FinO, while the *K*_d_ value for binding to ProQ^NTD^ was estimated as higher than 200 nM. The data shown for *malM*-3′ are the same as in Figure 1. Gels corresponding to the data in the plots are shown in Supplemental Figure S7. Average *K*_d_ values are shown in Table 1.

To dissect which specific sequence elements within the transplanted sequence of *malM*-3′ are responsible for differential recognition of this RNA by ProQ and FinO, we designed two *malM*-3′ mutants, in which only the nucleotides closest to the hairpin were substituted for such nucleotides as are present in corresponding positions in FinP (Fig. 3A). The first of these mutants, named *malM*-A-GU_6_, had each of the nucleotides directly adjacent to the terminator hairpin on its 5′ and 3′ side substituted for opposing A and G residues, respectively. The second mutant, named *malM*-A-GAU_5_, additionally had a uridine in the second position on the 3′ side of the hairpin substituted for adenosine. Hence, the *malM*-A-GU_6_ mutant had an A-G mismatch below the terminator hairpin the same as in FinP, while the *malM*-A-GAU_5_ mutant additionally had a C-A mismatch in the second position below the hairpin the same as in FinP (Figs. 1, 3A). The data showed that introducing the A-G mismatch into the *malM*-3′-A-GU_6_ mutant did not markedly affect its binding to ProQ^NTD^, which had a low nanomolar affinity, although the binding saturated at below 60%, as compared to 80% observed for unmodified *malM*-3′ (Fig. 3B; Table 1; Supplemental Fig. S7A). On the other hand, introducing the A-G mismatch very strongly improved the binding of FinO, because the affinity of the *malM*-3′-A-GU_6_ mutant to FinO was similar as that of FinP RNA (Fig. 3B; Table 1; Supplemental Fig. S7B). The additional substitution introducing the A residue in the second position on the 3′ side of the terminator hairpin had a strong detrimental effect on the binding of the resulting *malM*-A-GAU_5_ mutant to ProQ^NTD^ (Fig. 3B; Supplemental Fig. S7A). This effect was not caused by the shortening of the 3′-terminal oligoU sequence by the purine substitutions, because when the oligoU tail was extended to 7 nt as in wt *malM*-3′ the binding of the resulting *malM*-A-GAU_7_ mutant to ProQ^NTD^ was not restored (Supplemental Fig. S8). On the other hand, the binding of *malM*-A-GAU_5_ to FinO was not further improved as compared to *malM*-A-GU_6_ (Fig. 3B; Supplemental Fig. S7B). Additionally, we measured the binding of *malM*-A-GU_6_ mutant to the full-length ProQ (Supplemental Fig. S9A). The data showed that this mutant bound weaker than *malM*-3′ also to the full-length ProQ. Hence, in the context of *malM*-3′ body, the presence of the A-G mismatch in the first position together with the C-A mismatch in the second position below the terminator hairpin disrupted the binding to ProQ^NTD^, while only the A-G mismatch in the first position was sufficient to rescue the binding to FinO.

### Dissection of FinP sequence elements, which determine the binding specificity toward FinO

**FIGURE 4.**
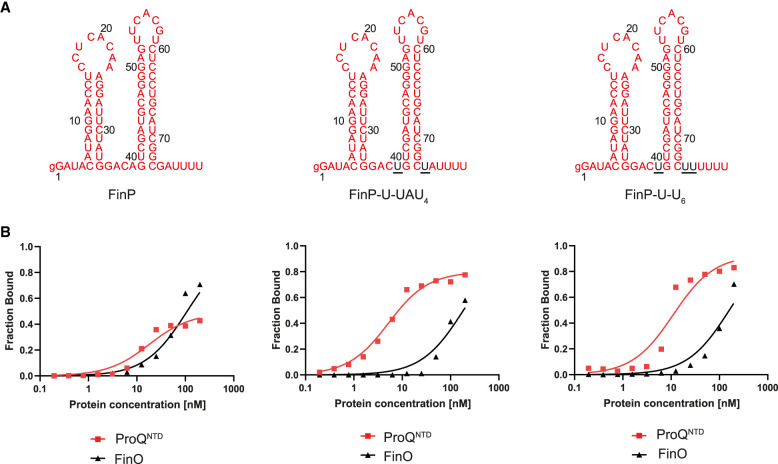
Comparison of FinP, FinP-U-UAU_4_, and FinP-U-U_6_ binding to ProQ^NTD^, and to FinO. (*A*) Secondary structures of FinP, FinP-U-UAU_4_, and FinP-U-U_6_, which were predicted using RNAstructure software (Reuter and Mathews 2010). The nucleotides from *malM*-3′ which were substituted into FinP are shown in black underlined font. The lowercase g denotes guanosine residue added on 5′ ends of RNA molecules to enable T7 RNA polymerase transcription. (*B*) The respective binding data for ProQ^NTD^ and FinO are shown on the graphs *below* each RNA. The fitting of the quadratic equation into FinP-U-UAU_4_ data provided a *K*_d_ value of 4.6 nM for binding to ProQ^NTD^, and 184 nM for binding to FinO. The fitting of FinP-U-U_6_ data using the quadratic equation provided a *K*_d_ value of 11 nM for binding to ProQ^NTD^, and 160 nM for binding to FinO. The data shown for FinP are the same as in Figure 1. Gels corresponding to the data in the plots are shown in Supplemental Figure S10. Average *K*_d_ values are shown in Table 1.

To explore whether the sequence elements at the two positions closest to the terminator hairpin of FinP are also involved in the differential recognition of this RNA by ProQ and FinO, we designed two mutants of FinP (Fig. 4; Table 1). The first mutant, named FinP-U-UAU_4_, only had both purines in the first positions below the terminator hairpin substituted with uridines, which are present in the corresponding locations in *malM*-3′ (Fig. 4A). The second mutant, named FinP-U-U_6_, additionally had the adenosine in the second position below the hairpin on the 3′ side replaced with a uridine, which is present in the corresponding position in *malM*-3′ (Fig. 4A). The data showed that the substitution of the A-G mismatch to the U-U mismatch in the FinP-U-UAU_4_ mutant was sufficient to improve its binding by ProQ^NTD^ with the *K*_d_ value in a low nanomolar range (Fig. 4B; Table 1; Supplemental Fig. S10A). On the other hand, these mutations had only a weak approximately twofold detrimental effect on the binding of this RNA to FinO (Fig. 4B; Table 1; Supplemental Fig. S10B). The additional substitution of adenosine to uridine in the second position on the 3′ side of the hairpin in FinP-U-U_6_ did not further affect the binding to either protein beyond the changes already caused by the substitution of the purine nucleotides in the FinP-U-UAU_4_ mutant with uridines (Fig. 4B; Table 1; Supplemental Fig. S10). To test whether the tight binding of FinP-U-U_6_ to ProQ^NTD^ was caused by the displacement of the purine nucleotides or by the resulting lengthening of the 3′-terminal oligoU sequence, we compared its binding with that of FinP-U-U_4_ mutant which had the number of 3′-terminal uridine residues the same as in FinP (Supplemental Fig. S11). The data showed that shortening the 3′ U tail to four residues in the FinP-U-U_4_ mutant did not weaken the binding of ProQ^NTD^, which suggests that the main reason for the improved binding of the FinP-U-U_6_ mutant to ProQ^NTD^ is the absence of the purine–purine mismatch neighboring the terminator hairpin, rather than the lengthening of the stretch of uridine residues at the 3′ end. Additionally, the data showed markedly weaker binding of the FinP-U-U_4_ mutant to FinO, which suggests that the binding of the FinO protein is more negatively affected than the binding of ProQ^NTD^ by the shortening of the total 3′ tail length in the context of the FinP body (Supplemental Fig. S11). When we additionally measured the binding of FinP-U-U_6_ mutant to full-length ProQ, the data showed that this mutant bound tighter than FinP also to the full-length ProQ (Table 1; Supplemental Fig. S9B). In summary, these data suggest that the presence of the A-G mismatch directly below the closing G-C base pair of FinP RNA terminator hairpin weakens its binding to the FinO domain of ProQ.

### The competition between ProQ and FinO for binding to *malM*-3′, FinP, and their mutants

To further explore how the sequence elements of *malM*-3′ and FinP affect their recognition by ProQ and FinO, we compared the binding of ProQ and FinO to these RNAs and their mutants, in reactions containing both proteins at the same concentration (Supplemental Fig. S12). The separation of RNA complexes with ProQ or FinO in the same reaction mixture was possible, because of different electrophoretic mobility of RNA-ProQ and RNA-FinO complexes. In this experiment, we compared the fractions of ^32^P-labeled RNA bound to ProQ and FinO, either in control reactions with each protein alone or in the competition reaction where both proteins were present at the same 100 nM concentration. At first, we compared the binding of ProQ and FinO to *malM*-3′ (Supplemental Fig. S12A). The data showed that in the reaction with both ProQ and FinO, the majority of RNA was bound to ProQ. Additionally, the fraction of *malM*-3′ bound to FinO was decreased in the competition reaction as compared to the control reaction with FinO only, which showed that ProQ outcompeted FinO from binding to *malM*-3′. Next, we compared the binding of ProQ and FinO to FinP (Supplemental Fig. S12D). The data showed that in the reaction with both ProQ and FinO, the majority of FinP RNA was bound to FinO. Additionally, there was a marked decrease of the fraction of FinP bound to ProQ in the competition reaction as compared to the control reaction with ProQ only, which supports the conclusion that FinO outcompeted ProQ from binding to FinP.

Next, we compared how introducing sequence elements from FinP into *malM*-3′ affects the competition between ProQ and FinO for binding to the resulting RNA mutants (Supplemental Fig. S12B,C). While similar fractions of *malM*-FinP chimera were bound to ProQ and FinO in control reactions, the majority of this RNA was bound by FinO in the competition reaction (Supplemental Fig. S12B). A similar result was observed in the reactions with *malM*-A-GAU_5_, where the majority of this RNA mutant was bound to FinO in the competition reaction, even though the fractions bound to each protein in the control reactions were similar (Supplemental Fig. S12C). These experiments showed that FinO also outcompeted ProQ in binding to both *malM*-FinP chimera and *malM*-A-GAU_5_ mutant.

Finally, we compared how transplanting sequence elements from *malM*-3′ into FinP affects the competition between ProQ and FinO for binding to the resulting RNA mutants (Supplemental Fig. S12E,F). In the competition reaction, similar fractions of FinP-*malM* were bound to ProQ and FinO, even though slightly larger fraction of this RNA chimera was bound to ProQ than FinO in the control reactions (Supplemental Fig. S12E). A similar result was observed in the reactions with FinP-U-U_6_, where similar fractions of FinP-U-U_6_ were bound to ProQ and FinO, while slightly larger fraction of this mutant was bound to ProQ than FinO in the control reactions (Supplemental Fig. S12F). However, in the competition reactions, the fractions of FinP-*malM* and FinP-U-U_6_ bound to ProQ (Supplemental Fig. S12E,F) are still markedly bigger than the fraction of FinP bound to ProQ (Supplemental Fig. S12D), which is consistent with the conclusion that transplanting the sequence elements from *malM*-3′ into FinP improves the recognition of the resulting RNA by ProQ.

### Sequence elements transplanted from FinP into the body of another ProQ ligand, *cspE*-3′ RNA, prevent the binding to ProQ^NTD^

**FIGURE 5.**
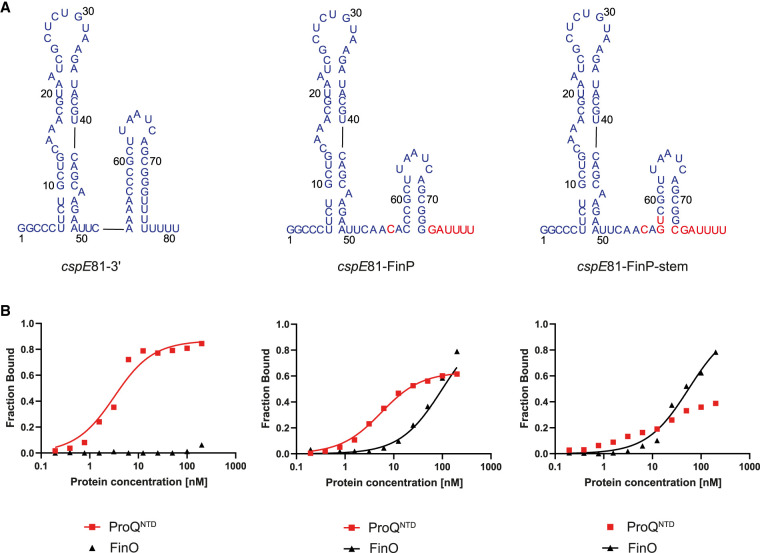
Comparison of *cspE*81-3′, *cspE*81-FinP chimera, and *cspE*81-FinP-stem chimera binding to ProQ^NTD^, and to FinO. (*A*) Secondary structures of *cspE*81-3′, *cspE*81-FinP chimera, and *cspE*81-FinP-stem chimera, which were predicted using RNAstructure software (Reuter and Mathews 2010). The sequences originating from *cspE*81-3′ are shown in dark blue font, and the sequences from FinP in red font. The lowercase g denotes guanosine residue added on 5′ ends of RNA molecules to enable T7 RNA polymerase transcription. (*B*) The respective binding data for ProQ^NTD^ and FinO are shown on the graphs *below* each RNA. The fitting of the quadratic equation into *cspE*81-3′ data provided a *K*_d_ value of 2.7 nM for binding to ProQ^NTD^, while the *K*_d_ value for binding to FinO was estimated as higher than 200 nM. The fitting of the quadratic equation into *cspE*81-FinP data provided a *K*_d_ value of 5.0 nM for binding to ProQ^NTD^, and 99 nM for binding to FinO. The fitting of the quadratic equation into *cspE*81-FinP-stem data provided a *K*_d_ value of 55 nM for binding to FinO, while the *K*_d_ value for binding to ProQ^NTD^ was estimated as higher than 200 nM. Gels corresponding to the data in the plots are shown in Supplemental Figure S13. Average *K*_d_ values are shown in Table 1.

The data presented above showed that transplanting sequence elements surrounding the terminator hairpin of FinP into the corresponding positions in *malM*-3′ body weakened its binding to ProQ^NTD^ and strengthened to FinO (Figs. 1–3; Table 1). However, *malM*-3′ belongs to only few RNAs among the top ligands of ProQ, which naturally contain a pyrimidine–pyrimidine mismatch immediately below the closing G-C or C-G base pair of the terminator hairpin (Supplemental Figs. S1, S2). The majority of top RNA ligands of ProQ have an A-U base pair in this position. An example is *cspE*-3′, which is among RNAs bound by ProQ identified in CLIP-seq and RIL-seq studies (Holmqvist et al. 2018; Melamed et al. 2020). The in vitro binding of *cspE*-3′ to *E. coli* ProQ and ProQ^NTD^ has been previously studied (Stein et al. 2020). To test if the binding specificity of 81 nt long *cspE*81-3′ is also dependent on sequences surrounding the transcription terminator, we transplanted the sequences surrounding the terminator hairpin from FinP into *cspE*-3′ (Fig. 5A). In the natural sequence of *cspE*-3′, the 4 nt 5′-adjacent to the terminator hairpin are all adenosines, and it has a 3′-terminal U_8_ sequence, of which four uridines nearest to the closing C-G base pair of the terminator hairpin are base-paired with opposing adenosines (Fig. 5A). The sequence elements transplanted from FinP into the *cspE*81-3′ body were the C residue in the second position on the 5′-side to the terminator hairpin, and the 3′-terminal GAU_4_ sequence (Fig. 2A). As a result, the *cspE*81-FinP chimera had the three nucleotides 5′-adjacent to the terminator hairpin and the whole 3′-terminal tail the same as in FinP.

The data showed that *cspE*81-3′ bound to ProQ^NTD^ with a *K*_d_ value in the low nanomolar range, which was even twofold tighter than that of *malM*-3′ (Fig. 5B; Table 1; Supplemental Fig. S13A). On the other hand, its binding to FinO protein was negligible with a maximum fraction bound of 10% at the 200 nM concentration of FinO (Fig. 5B; Table 1; Supplemental Fig. S13B). The introduction of substitutions, which made the sequence surrounding the terminator in the *cspE*81-FinP chimera the same as in FinP, resulted in the moderate, twofold weakening of *cspE*81-FinP binding to ProQ^NTD^, and also resulted in a lower maximum fraction bound. On the other hand, these substitutions restored the binding of *cspE*81-FinP RNA to FinO to the affinity similar as that of FinP RNA. Because we noted that the two base pairs at the base of the terminator hairpin differ between *cspE*81-3′ and FinP, we next made additional substitutions into *cspE*81-3′ to ensure that in the resulting *cspE*81-FinP-stem chimera, not only the surrounding sequence but also the two lowest base pairs of terminator hairpin are the same as in FinP (Fig. 5A). While these substitutions did not markedly affect the binding of *cspE*81-FinP-stem chimera to FinO, they resulted in further weakening of its binding to ProQ^NTD^ (Fig. 5B; Table 1; Supplemental Fig. S13). We also measured the binding of *cspE*81-3′ and the *cspE*81-FinP-stem chimera to ProQ. The data showed that this mutant bound weaker than *cspE*81-3′ also to the full-length ProQ (Table 1; Supplemental Fig. S9C).

Additionally, we compared the binding of *cspE*81-3′ and *cspE*81-FinP-stem chimera to ProQ and FinO present at equal concentrations in the reaction (Supplemental Fig. S14A,B). The data showed that almost all of *cspE*81-3′ was bound to ProQ, in the reactions where both proteins were present. The same difference was observed in control reactions with either protein alone. On the other hand, a bigger fraction of the *cspE*81-FinP-stem chimera was bound to FinO than ProQ. Additionally, the fraction of *cspE*81-FinP-stem bound to ProQ was decreased in the competition reaction as compared to the control reaction with ProQ only, which showed that FinO outcompeted ProQ from binding the *cspE*81-FinP-stem chimera. These data confirm the contribution of the nucleotides at the base of terminator hairpins of *cspE*81-3′ and FinP in their recognition by ProQ and FinO.

In summary, these data show that in the context of the *cspE*81-3′ body, both the sequence surrounding the terminator hairpin and the two lowest base pairs of the hairpin affect the recognition of *cspE*81-3′ by ProQ^NTD^, while the sequence surrounding the terminator hairpin is sufficient to ensure the recognition by FinO. This suggests that while the recognition of *malM*-3′ and *cspE*81-3′ by the FinO domain of ProQ and by FinO is dependent on the features of the junction between the terminator hairpin and the surrounding sequence, the details of the recognition differ between these two RNAs.

### Sequence elements transplanted from another FinO ligand, RepX RNA, into the body of *malM*-3′ weaken the binding to ProQ^NTD^

**FIGURE 6.**
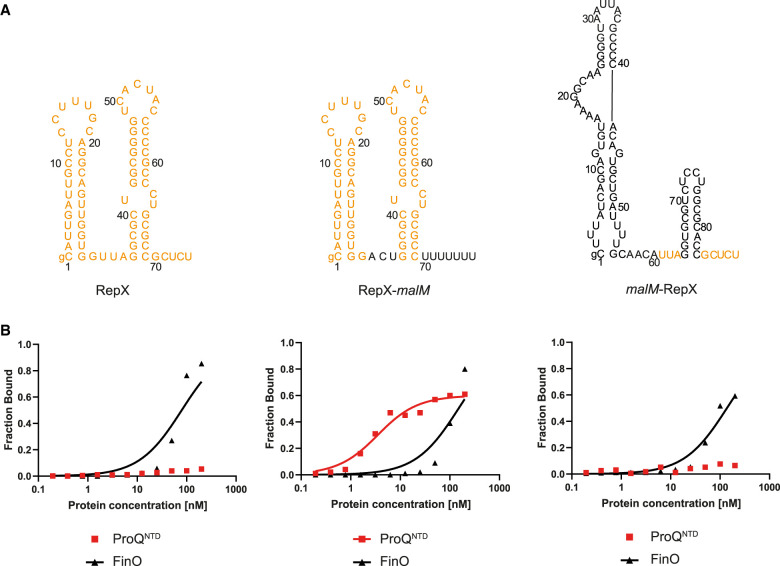
Comparison of RepX, RepX-*malM* chimera, and *malM*-RepX chimera binding to ProQ^NTD^, and to FinO. (*A*) Secondary structures of RepX, RepX-*malM* chimera, and *malM*-RepX chimera, which were predicted using RNAstructure software (Reuter and Mathews 2010). The sequences originating from RepX are shown in orange font, and the sequences from *malM*-3′ in black font. The lowercase g denotes guanosine residue added on 5′ ends of RNA molecules to enable T7 RNA polymerase transcription. (*B*) The respective binding data for ProQ^NTD^ and FinO are shown on the graphs *below* each RNA. The fitting of the quadratic equation into RepX data provided a *K*_d_ value of 79 nM for binding to FinO, while the *K*_d_ value for binding to ProQ^NTD^ was estimated as higher than 200 nM. The fitting of the quadratic equation into RepX-*malM* data provided a *K*_d_ value of 3.0 nM for binding to ProQ^NTD^, and 148 nM for binding to FinO. The fitting of the quadratic equation into *malM*-RepX data provided a *K*_d_ value of 137 nM for binding to FinO, while the *K*_d_ value for binding to ProQ^NTD^ was estimated as higher than 200 nM. Gels corresponding to the data in the plots are shown in Supplemental Figure S15. Average *K*_d_ values are shown in Table 1.

Because recent studies in *S. enterica* showed that the F-like plasmid FinO protein binds specifically not only to FinP but also to another RNA, named RepX (El Mouali et al. 2021a), we tested if sequence elements surrounding the terminator hairpin of RepX serve a similar role in determining RNA recognition by FinO as we observed for FinP (Figs. 1–3; Table 1). For this, we designed two chimeric constructs. In one, the sequence elements surrounding the terminator hairpin in *malM*-3′ were transplanted into RepX (Fig. 6A). The sequence elements transplanted into the RepX-*malM* chimera from *malM*-3′ were the ACU sequence on the 5′ side of the terminator hairpin, and the U_7_ tail on the 3′ side of the hairpin (Fig. 6A). In the other chimera, the sequence elements surrounding the terminator hairpin in RepX were transplanted into *malM*-3′ (Fig. 6A). The sequence elements transplanted into the *malM*-RepX chimera from RepX were the UUA sequence on the 5′ side of the terminator hairpin, and the GCUCU tail on the 3′ side of the hairpin (Fig. 6A). Of note, the nucleotide residues nearest to the closing base pair of the terminator hairpin of RepX—A and G—are the same as in FinP.

The data showed that the binding of wt RepX chimera to ProQ^NTD^ was almost undetectable up to 200 nM concentration of the protein (Fig. 6B; Table 1; Supplemental Fig. S15A), which is consistent with the weak binding of FinP to ProQ and ProQ^NTD^ (Figs. 1B,C,E, 2B; Table 1; Supplemental Fig. S5). At the same time, RepX bound the FinO protein with a *K*_d_ value of 61 nM (Fig. 6B; Table 1; Supplemental Fig. S15B), which is similar to the affinity of FinP binding to FinO (Fig. 1D,E; Table 1). In contrast, the RepX-*malM* chimera bound strongly to ProQ^NTD^ with a *K*_d_ value similar to that of *malM*-3′, although its binding to FinO was not much affected by the transplantation (Fig. 6B; Table 1; Supplemental Fig. S15B). This showed that removing the RepX sequence adjacent to the terminator hairpin enabled the tight binding of RepX-*malM* chimera to ProQ^NTD^. When the binding of the reverse chimera, *malM*-RepX, was measured the data showed that its binding to ProQ^NTD^ was almost undetectable, which is similar to RepX (Fig. 6B; Table 1; Supplemental Fig. S15A). The data also showed that *malM*-RepX chimera bound weaker to full-length ProQ than *malM*-3′ (Table 1; Supplemental Fig. S9D). On the other hand, *malM*-RepX chimera bound FinO with a *K*_d_ value of 180 nM, which is only twofold weaker than for RepX (Fig. 6B; Table 1; Supplemental Fig. S15B). We also compared the binding of RepX and the *malM*-RepX chimera to ProQ and FinO, in reactions where both proteins were present (Supplemental Fig. S14C,D). The data showed that almost all of RepX was bound to FinO in the competition reactions, which was similar as in the control reactions. Also, a bigger fraction of the *malM*-RepX chimera was bound to FinO than ProQ. Additionally, the fraction of the *malM*-RepX chimera bound to ProQ was decreased in the competition reaction as compared to the control reaction with ProQ only, which showed that FinO outcompeted ProQ from binding the *malM*-RepX chimera. Overall, these data showed that sequence elements surrounding the intrinsic terminator hairpin in RepX have a similar role as the corresponding sequence elements in FinP, because they function to weaken RNA binding by ProQ and ProQ^NTD^.

## DISCUSSION

The data presented here show that the sequences surrounding the transcription terminator hairpin contribute not only to the strength of binding of FinO-domain proteins to their RNA ligands, but also to the discrimination among RNAs by these proteins (Figs. 1–6; Table 1; Supplemental Figs. S12, S14). The recent crystal structure of the FinO domain of *L. pneumophila* RocC protein in complex with RocR RNA showed in molecular detail how the lower part of RocR hairpin and its 3′ polypyrimidine tail interact with amino acid residues on the concave face of the FinO domain of RocC (Kim et al. 2022). Additionally, several in vitro binding studies showed the importance of the lower part of the terminator hairpin and adjacent single-stranded sequences for tight RNA binding by *E. coli* ProQ (Chaulk et al. 2011; Stein et al. 2020, 2023) and FinO (van Biesen and Frost 1994; Jerome and Frost 1999; Arthur et al. 2011). Here, we observed that transplanting sequences surrounding the terminator hairpins between respective RNA ligands of each protein changed the binding affinities of these RNAs to ProQ, ProQ^NTD^, and FinO in agreement with the origin of the transplanted sequence (Figs. 1–6; Table 1). This effect was also seen in reactions containing both proteins (Supplemental Figs. S12, S14). Hence, the data presented here expand the role of this RNA region by showing that the features of RNA sequences surrounding the terminator hairpin contribute to the discrimination between RNAs by *E. coli* ProQ and FinO.

Why does ProQ not recognize the natural RNA ligands of the FinO protein, FinP, and RepX? The unusual feature of both FinP and RepX is the presence of a purine–purine mismatch immediately below the closing G-C base pair of the terminator hairpin (Supplemental Fig. S3). On the other hand, most of the top RNA ligands of ProQ have extended A-U base-pairing below the terminator stem (Supplemental Figs. S1, S2). This group includes *cspE*-3′. The continued base-pairing involving the upstream A-rich sequence is a feature of *cspE* transcription terminators also in other bacteria (Supplemental Fig. S16). Although A-U base-pairing is not predicted for *malM*-3′, which contains a pyrimidine–pyrimidine mismatch below the terminator, the sequence on the 5′ side of the terminator contains several adenosines, which suggests that less stable base-pairing in this region could still form. The presence of U-U mismatch is also a feature of the base of *malM* transcription terminators in other bacteria (Supplemental Fig. S16). Notably, a pyrimidine–pyrimidine mismatch in the first position below the terminator hairpin, which conformed to the A-type double helix geometry, was observed in the structure of *L. pneumophila* RocR RNA with RocC protein (Kim et al. 2022). This suggests that the RNA-binding site of the FinO domain of ProQ can accommodate RNAs, which contain A-U base pairs and pyrimidine–pyrimidine mismatches below the closing base pair of the terminator. We hypothesize that the reason why the sequences surrounding the terminators of FinP and RepX weaken their binding by ProQ (Figs. 2, 3, 6; Table 1) is that the purine–purine mismatch together with neighboring sequence elements disrupt the double-helical structure of this region in a way, which is not compatible with its recognition by the FinO domain of ProQ. Additionally, as there are many more RNAs bound by ProQ than by FinO, it is possible that the competition among RNAs could serve as an additional mechanism preventing the binding of FinO-specific RNAs to ProQ in the bacterial cell.

How do the sequences surrounding terminators of FinP and RepX ensure their preferred binding by FinO? The role of such features in RNA recognition by FinO is expected to be especially important because of the large pool of RNAs specific for ProQ. Introducing the sequences from FinP or RepX into the context of *malM*-3′ or *cspE*-3′ strengthened the binding of these RNAs to the FinO protein (Figs. 2, 3, 5, 6; Table 1). This suggests that the sequences naturally surrounding the terminator of FinP and RepX introduced into chimeric RNAs such features that are recognized by FinO. On the other hand, when the corresponding sequences from *malM*-3′ were introduced into FinP or RepX it had only a moderate detrimental effect on the binding by FinO (Figs. 4, 6; Table 1). It was previously proposed that the RNA strands surrounding the terminator of FinP are separated in the complex of FinP with FinO (Arthur et al. 2011). We hypothesize that the natural sequence of FinP, including the purine–purine mismatch, helps to facilitate the strand separation, which is why introducing such sequence into *malM*-3′ or *cspE*-3′ strengthens the binding. On the other hand, the moderate detrimental effect of replacing the natural sequence of FinP or RepX with that from *malM*-3′ could suggest either that the strands originating from this RNA are sufficiently separated in the context of unnatural RNA body to enable the binding by FinO, or that other parts of the FinP or RepX structure also contribute to the RNA binding by FinO. In support of the latter hypothesis, it was previously observed that the sole terminator hairpin of FinP, devoid of surrounding single-stranded regions, was still able to bind FinO, albeit much weaker than the intact RNA (Jerome and Frost 1999). Regardless of the detailed explanation, we hypothesize that the differences in sequences around the terminator hairpins of natural RNA ligands of ProQ and FinO lead to differences in local RNA structure, thus affecting RNA recognition by each protein.

What properties of distinct FinO-domain proteins make them capable of differently recognizing the same RNAs? The X-ray structure of a complex of *L. pneumophila* RocC protein with RocR RNA showed that the double-helical stem of RocR terminator hairpin and the end of its 3′ tail were recognized by two distinct regions of the FinO domain of RocC (Kim et al. 2022). In this structure, the two terminal nucleotides of the 3′ polypyrimidine tail were bound by a conserved group of residues including a tyrosine and an arginine corresponding to *E. coli* ProQ residues Y70 and R80. However, because these amino acids are the same in *E. coli* ProQ and F-plasmid FinO protein, they would not be expected to be directly responsible for different RNA recognition by ProQ and FinO. On the other hand, the lower part of the double-helical stem of the RocR terminator hairpin is bound by a set of hydrogen-bond forming amino acids in the N-terminal part of α-helix 5, which was named the α-helical N-cap motif (Kim et al. 2022). The N-cap motif of RocC consists of two serines, two lysines, and an arginine of α-helix 5 (S70, K71, S72, K74, and R76), which are within hydrogen-bonding distance to nonbridging oxygens of the phosphates of the 3′ strand of the terminator stem of RocR (Kim et al. 2022). As these amino acid side chains contact the base of the terminator hairpin, and there are differences in the sequences forming corresponding α-helices between ProQ and FinO, it is possible that this region could be responsible for differential recognition of RNA ligands by ProQ and FinO.

**FIGURE 7.**
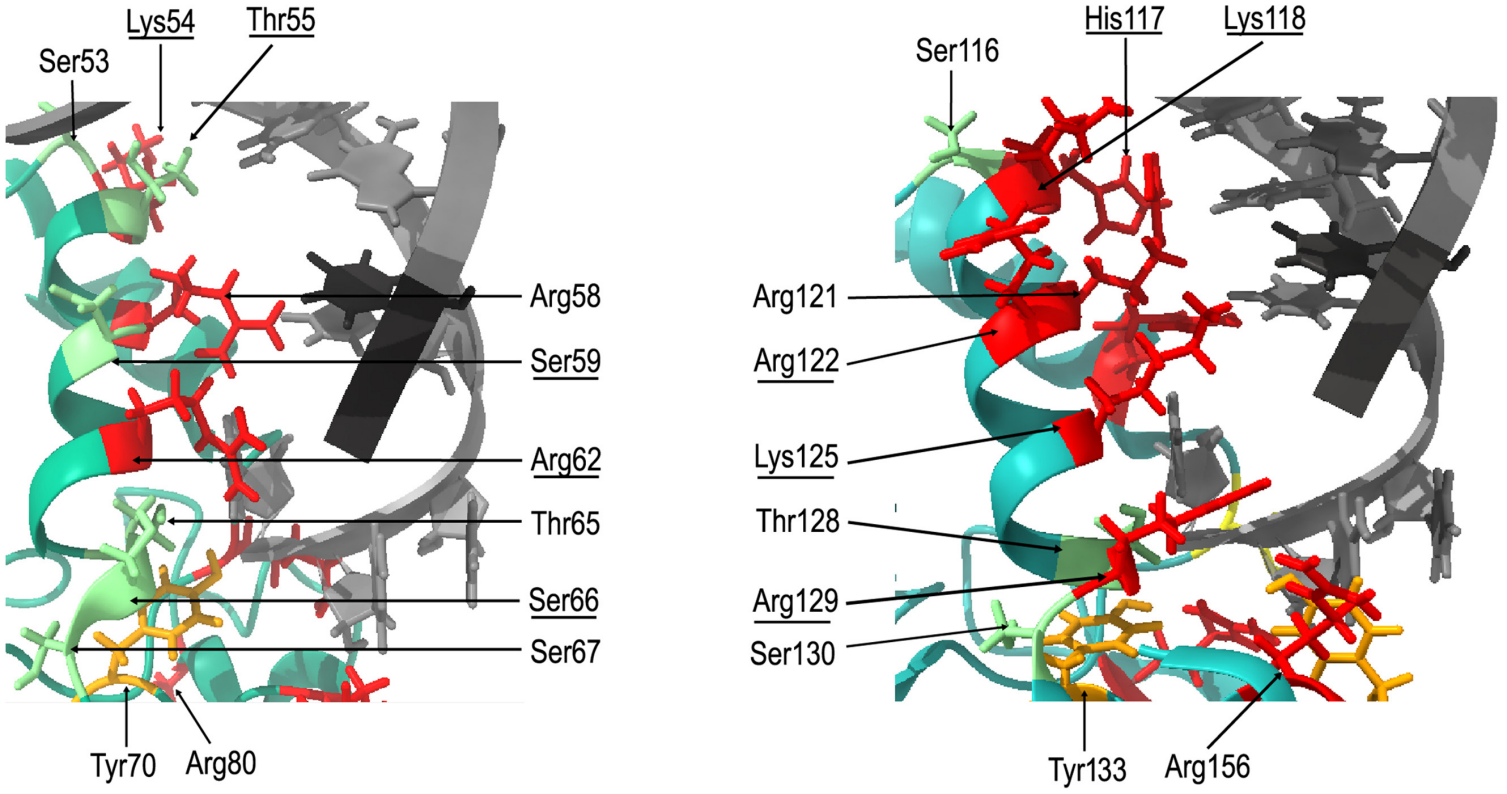
The modeling of RNA-binding surfaces of *E. coli* ProQ and F-like plasmid FinO. The figure shows the α-helix 3 and surrounding region from *E. coli* ProQ (*left*) and the corresponding α-helix 4 from F-like plasmid FinO (*right*) with those amino acid residues marked, which side chains are directed toward the modeled location of RNA helix. The modeling of interactions was done using Chimera X (Pettersen et al. 2021) by aligning the NMR structure of the FinO domain of *E. coli* ProQ (Gonzalez et al. 2017) and the X-ray structure of the F-like plasmid FinO protein (Ghetu et al. 2000) with the X-ray structure of the FinO domain of *L. pneumophila* RocC in complex with the terminator hairpin of RocR RNA (Kim et al. 2022). The side chains of amino acid residues located in the corresponding positions of both proteins are marked in color, with arginine, lysine, and histidine residues marked in red, serine and threonine in green, and tyrosine in orange. The descriptions of corresponding amino acids are located in corresponding places on the figure. Those amino acids which are different, but located in corresponding positions, are marked by underlining. The structure of the *L. pneumophila* RocR hairpin is shown in gray.

Because the structures of the complexes of *E. coli* ProQ and FinO with their RNA ligands are not known, we overlayed the NMR structure of the FinO domain of ProQ (Gonzalez et al. 2017) and the X-ray crystal structure of FinO (Ghetu et al. 2000) on the structure of the complex of RocC with RocR (Kim et al. 2022) to visualize the surfaces of α-helices of ProQ and FinO corresponding to RocC α-helix 5, which would likely be exposed toward bound RNA (Fig. 7). For comparison, we also modeled these interactions by overlaying the ColabFold-generated structures of the FinO domain of ProQ protein and the FinO protein on the structure of the complex of RocC with RocR (Supplemental Fig. S17; Kim et al. 2022; Mirdita et al. 2022). The orientations of amino acid side chains toward RNA were similar in the models based on ColabFold-generated structures as in models based on experimentally obtained structures (Fig. 7; Supplemental Fig. S17). The analysis of the modeled interactions showed that the N-cap motif in α-helix 3 of ProQ is quite similar to that of RocR, because it contains hydrogen-bonding residues in corresponding positions (S53, K54, R58, and R62) (Fig. 7; Supplemental Fig. S17). The importance of K54, R58, and R62 for RNA binding by *E. coli* ProQ has previously been shown by mutagenesis experiments in vivo and in vitro (Pandey et al. 2020; Stein et al. 2023). On the other hand, in the FinO protein the surface of the corresponding α-helix 4, from which hydrogen-bonding residues are directed toward modeled RNA molecules, is more extended and covers the whole length of this α-helix. It includes seven hydrogen-bonding residues, which side chains are oriented toward RNA double helix (H117, K118, R121, R122, K125, and R129) (Fig. 7; Supplemental Fig. S17). The importance of R121 and K125 for RNA binding by FinO has already been shown using cysteine substitutions and cross-linking (Ghetu et al. 2002). The amino acid residues K125 and R129 of FinO are located in the C-terminal part of α-helix 4, which, in the model of the interaction shown in Figure 7, is close to the base of the terminator hairpin. Interestingly, in ProQ the residue homologous to FinO K125 is arginine (R62), and to FinO R129 is serine (S66). These differences suggest a possibility of different interactions between the RNA-binding pocket of the FinO domain and the base of the terminator hairpin in ProQ and FinO. Overall, the comparison of distributions of hydrogen-bonding amino acids in ProQ α-helix 3 and corresponding FinO α-helix 4 shows marked differences, which could form the basis of unique interactions that enable each protein to differently recognize RNA molecules.

## MATERIALS AND METHODS

### Preparation of RNAs

The DNA templates for in vitro transcription were obtained by Taq polymerase extension of chemically synthesized overlapping oligodeoxyribonucleotides (Sigma-Aldrich and Metabion) (Supplemental Table S1). RNA molecules used in this study were obtained using in vitro transcription with T7 RNA polymerase as described (Milligan et al. 1987; Olejniczak 2011). After transcription, RNA molecules were purified using 8 M urea polyacrylamide gel electrophoresis. RNAs were 5′-^32^P-labeled using T4 polynucleotide kinase (Thermo Scientific) and γ-^32^P ATP (Hartmann Analytic), which was followed by phenol–chloroform extraction, denaturing gel electrophoresis, and ethanol precipitation. 5′-^32^P-labeled RNAs were stored at −20°C as 200 nM solutions.

### Expression and purification of proteins

The sequences of *E. coli* ProQ protein, and its 130 aa long N-terminal domain were cloned into pET-15b vector (Novagen), and purified as previously described (Stein et al. 2020). To obtain the FinO protein the overexpression construct was prepared by cloning the coding sequence of *finO* into pET-15b vector (Novagen) using BamHI restriction site (Supplemental Table S2). The coding sequence was obtained by amplification from the template of *finO* construct in pGEX-KG vector, which was a kind gift of Professor Mark Glover (University of Alberta). In the expression construct the coding sequence was preceded by His_6_-tag and TEV protease recognition sequence (ENLYFQ↓S). The construct was overexpressed in BL21 Δ*hfq E. coli* strain (a kind gift of Professor Agnieszka Szalewska-Pałasz, University of Gdansk) and purified as previously described for *E. coli* ProQ (Stein et al. 2020). The molecular mass of the purified protein was determined by MALDI-TOF as 21,362.1 Da, which is close to the calculated mass of 21,366.6 Da. The samples were stored in a buffer consisting of 50 mM Tris, pH 7.5, 300 mM NaCl, 10% glycerol, and 1 mM EDTA, at −80°C, in 5 and 10 μL aliquots, and used without refreezing.

### Gelshift assays of RNA binding by ProQ, ProQ^NTD^, and FinO

RNA binding to ProQ, ProQ^NTD^, or FinO proteins was measured using electrophoretic mobility shift assays. The concentration series of ProQ, ProQ^NTD^, or FinO were made by twofold sequential dilutions. RNAs were denatured at 90°C for 2 min, followed by refolding on ice for 5 min. To initiate the binding reaction a ^32^P-labeled RNA (1 nM final concentration) was mixed with indicated final concentrations of ProQ, ProQ^NTD^, or FinO in the binding buffer (150 mM NaCl, 25 mM Tris-HCl pH 7.5, 5% glycerol, 1 mM MgCl_2_) and incubated for 30 min at room temperature. Incubation was performed in low-protein binding microplates, additionally pretreated with a solution containing 0.0025% bovine serum albumin. After 30 min of incubation, 5 µL reaction aliquots were loaded onto a native 6% polyacrylamide gel (19:1), and run in 0.5× TBE at 4°C. Gels were dried using a vacuum dryer, and exposed to phosphor screens, followed by data quantification using a phosphorimager and MultiGauge software (Fuji FLA-5000). The equilibrium dissociation constant (*K*_d_) values were obtained by fitting the quadratic equation into binding data using GraphPad Prism software as described (Stein et al. 2020). Average *K*_d_ values with standard deviations were calculated from at least three independent experiments.

## SUPPLEMENTAL MATERIAL

Supplemental material is available for this article.

## Figures and Tables

**Maria D. Mamońska:**
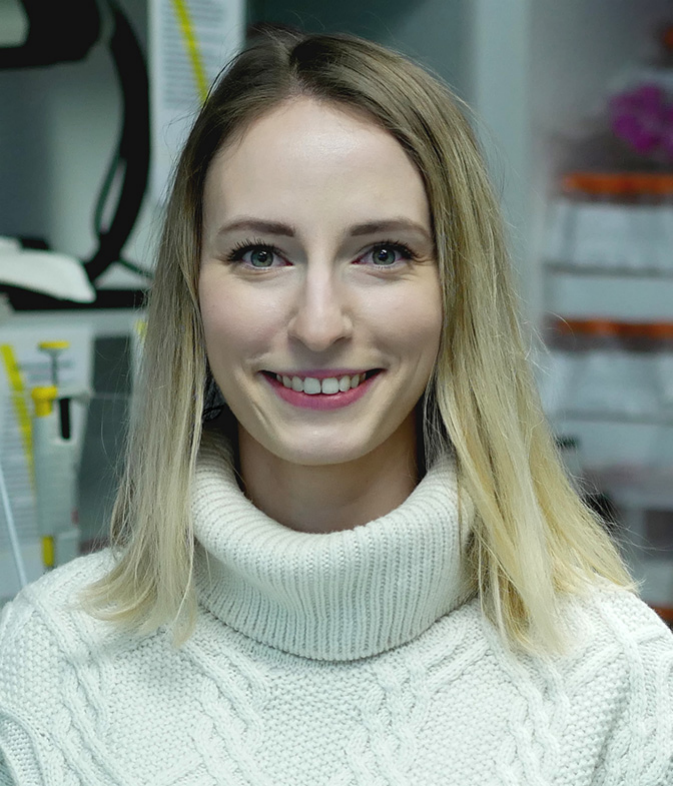


## MEET THE FIRST AUTHOR

***Meet the First Author(s)* is an editorial feature within *RNA*, in which the first author(s) of research-based papers in each issue have the opportunity to introduce themselves and their work to readers of *RNA* and the RNA research community. Maria D. Mamońska is the first author of this paper, “Different RNA recognition by ProQ and FinO depends on the sequence surrounding intrinsic terminator hairpins.” Maria is a PhD student at the Institute of Molecular Biology and Biotechnology at Adam Mickiewicz University in Poznań, Poland. Her research focuses on the competition among RNA-binding proteins ProQ, FinO, and Hfq in regulating gene expression in *Escherichia coli*.**

**What are the major results described in your paper and how do they impact this branch of the field?**

In our paper, we present significant findings regarding the RNA-binding specificity of the ProQ and FinO proteins in *Escherichia coli*. We show that the RNA sequence elements surrounding the intrinsic terminator hairpin play a crucial role in how ProQ and FinO discriminate between their RNA targets. These findings help advance our understanding of the molecular mechanisms behind RNA-binding protein interactions, which are crucial for regulating gene expression in bacteria.

**What led you to study RNA or this aspect of RNA science?**

My interest in molecular biology began with a strong curiosity about how organisms function at the molecular level. During my master's thesis, I explored the complex world of RNA and its interactions with proteins, which helped me realize its key role in regulating gene expression. I was fascinated by how small changes in RNA sequences can have a big impact on protein binding and cellular behavior. I believe understanding these interactions could offer valuable insights into various cellular processes.

**What are some of the landmark moments that provoked your interest in science or your development as a scientist?**

A turning point that provoked my interest in science was taking a course on microbiomes during my undergraduate studies. I became fascinated by the critical role that diverse microbial communities play in influencing health and disease.

**If you were able to give one piece of advice to your younger self, what would that be?**

If I could give one piece of advice to my younger self, it would be to embrace curiosity and experimentation without fearing failure. In molecular biology, obstacles are common, and each setback offers valuable lessons. Every experiment, no matter the outcome, plays a crucial role in expanding your understanding of biological processes.
